# A spatial–microbial–metabolic–immune framework for high-risk oral potentially malignant disorders: an evidence-ranked review for malignant transformation risk stratification

**DOI:** 10.3389/fcimb.2026.1928595

**Published:** 2026-09-15

**Authors:** Shaofu Yan, Yanxin Zhang, Wenqi Tan, Jiehua Guo, Jinyan Si, Hongjiao Li, Jing Shi

**Affiliations:** 1 Shanxi Children's Hospital, Shanxi Women and Children Health Hospital, Taiyuan, Shanxi, China; 2 Shanxi Oral Health Prevention and Control Technology Innovation Center, Taiyuan, Shanxi, China; 3 Shanxi Medical University School and Hospital of Stomatology, Taiyuan, Shanxi, China; 4 The Fifth Clinical Medical College of Shanxi Medical University, Taiyuan, Shanxi, China

**Keywords:** contamination-aware microbial localization, host–microbe interaction, immunometabolism, oral epithelial dysplasia, oral microbiome, oral potentially malignant disorders, risk stratification, spatial multi-omics

## Abstract

The oral mucosa is a microbe-exposed barrier ecosystem in which surface biofilms, epithelial differentiation programs, microbial products, metabolic stress, stromal remodeling, and immune surveillance are spatially organized. Although oral microbiome studies have associated dysbiosis with oral potentially malignant disorders (OPMDs) and oral squamous cell carcinoma (OSCC), most evidence remains saliva-based, rinse-based, swab-based, tissue-homogenate-based, or taxon-centered. These approaches can identify disease-associated microbial patterns but cannot determine whether microbial signals are locally aligned with epithelial, metabolic, stromal, and immune changes within the same mucosal microdomains. Here, we propose a spatial–microbial–metabolic–immune (SMMI) framework as an evidence-ranked and testable approach for studying malignant transformation risk in high-risk OPMDs. The acronym denotes four analytical dimensions—spatial organization, microbial signals, metabolic mediation, and immune remodeling—all interpreted within oral host tissue. The framework asks whether dysbiosis-associated microbial signals, epithelial barrier-response states, metabolic stress, stromal remodeling, macrophage-centered immunoregulation, altered epithelial immune visibility, and T-cell positioning become locally aligned within candidate mucosal microdomains. SMMI is a conceptual and validation framework rather than an established causal mechanism, fixed anatomical structure, or immediate therapeutic target. This article is an evidence-ranked conceptual review rather than a systematic review. We distinguish direct human OPMD evidence from OSCC-derived inference, preclinical perturbation data, broader cancer biology, methods papers, and speculative but testable hypotheses. We outline a modular validation roadmap that proceeds from pathology-anchored sampling and contamination-aware microbial localization to hypothesis-selected spatial modules, targeted functional testing, and longitudinal clinical validation. The near-term translational goal is improved risk stratification and risk-adapted surveillance, rather than ecological intervention. By reframing high-risk OPMDs as spatial oral microbe–host immune-metabolic ecosystems, the SMMI framework provides a cautious structure for hypothesis generation, spatial validation, and clinically relevant risk modeling.

## 1 Introduction: high-risk OPMDs as a spatial oral microbe–host barrier model

Oral microbiome research has substantially expanded understanding of oral potentially malignant disorders (OPMDs) and oral squamous cell carcinoma (OSCC). Many studies have reported disease-associated microbial shifts in saliva, oral rinse, lesion swabs, mucosal scrapings, and tissue specimens, often involving anaerobic, inflammation-associated, periodontal, fungal, or polymicrobial patterns (Amer et al., 2017; Lamont et al., 2018; Pietrobon et al., 2021; Radaic and Kapila, 2021; Herreros-Pomares et al., 2023; Wright et al., 2023; Galvin et al., 2025). However, most microbiome evidence remains patient-level, surface-level, lesion-level, or bulk-tissue-based. These approaches identify microbial associations but cannot determine whether microbial signals are spatially adjacent to dysplastic epithelial states, metabolic stress, stromal remodeling, or immune-cell restriction within the same mucosal microdomains.

This limitation matters because oral mucosa is not a uniform surface. It is a microbe-exposed stratified barrier ecosystem in which surface biofilms, suprabasal differentiation, basal epithelial renewal, basement membrane integrity, lamina propria architecture, vascular structures, and immune-cell positioning are organized across interacting compartments (Zijnge et al., 2010; Mark Welch et al., 2016; Moutsopoulos and Konkel, 2018; Pelaez-Prestel et al., 2021; Senel, 2021). Microbial products may contact the epithelial surface; epithelial injury may reshape local microbial ecology; inflammatory responses may alter differentiation and metabolic stress; and immune cells may be recruited, restrained, or functionally remodeled in restricted regions. Oral malignant transformation should therefore not be understood only as a whole-lesion molecular event or a list of dysbiosis-associated taxa.

High-risk OPMDs provide a clinically accessible window for testing this possibility. OPMDs are heterogeneous oral mucosal disorders associated with increased oral cancer risk, including oral leukoplakia, erythroplakia, erythroleukoplakia, proliferative verrucous leukoplakia, oral submucous fibrosis, and selected oral lichenoid disorders (Speight et al., 2018; Aguirre-Urizar et al., 2021; Warnakulasuriya et al., 2021; Mohideen et al., 2025). These entities differ in phenotype, site, exposure background, inflammation, microbial context, and malignant transformation risk. The transition from high-risk OPMDs to early OSCC is therefore a useful model for asking whether spatially organized microbe–host interactions help explain lesion-level risk heterogeneity.

Histopathological assessment of oral epithelial dysplasia remains the clinical anchor of OPMD diagnosis and risk evaluation (Kujan et al., 2007; Ranganathan and Kavitha, 2019; Wils et al., 2020). Its predictive precision is limited by sampling, intralesional heterogeneity, interobserver variability, and divergent outcomes among morphologically similar lesions (Kujan et al., 2007; Schaaij-Visser et al., 2010; Farah et al., 2019; Ranganathan and Kavitha, 2019; Wils et al., 2020; Mahmood et al., 2023; Shephard et al., 2024). These limitations do not diminish histopathology. Rather, they indicate that morphology alone may not resolve how epithelial risk is spatially coupled to microbial exposure, metabolic stress, stromal remodeling, and immune architecture. A microbiologically informed spatial framework should complement conventional pathological assessment.

This Review proposes a spatial–microbial–metabolic–immune (SMMI) framework, in which spatial organization, microbial signals, metabolic mediation, and immune remodeling are interpreted within oral host tissue. It asks whether microbial signals, epithelial barrier-response states, metabolic pressures, stromal remodeling, macrophage-centered immunoregulation, altered epithelial immune visibility, and T-cell positioning become locally aligned within discrete mucosal regions. Such alignment nominates candidate SMMI microdomains and requires longitudinal, functional, and outcome-linked validation.

What this Review adds to oral microbe–host research. This Review reframes high-risk OPMDs as a model for studying spatial oral microbe–host interactions before overt invasion. It moves beyond a single-driver dysbiosis model by distinguishing microbial drivers, passengers, risk markers, and ecological amplifiers; emphasizes contamination-aware microbial localization; and integrates microbial signals with epithelial barrier states, metabolic mediation, stromal remodeling, and immune architecture. Direct human OPMD evidence is prioritized where available, while OSCC, preclinical, and broader cancer data are used to generate testable hypotheses. The near-term goal is risk stratification and risk-adapted surveillance.

## 2 Definitions, scope and evidence hierarchy

### 2.1 High-risk OPMDs and early OSCC as the clinical window

OPMDs comprise heterogeneous oral mucosal abnormalities associated with increased oral cancer risk. Here, high-risk OPMDs refers to lesions with clinical, histopathological, or biological features that justify intensified surveillance, including leukoplakia with moderate or severe dysplasia, erythroplakia, erythroleukoplakia, non-homogeneous red-white lesions, proliferative verrucous leukoplakia, and persistent, multifocal, or extensive mucosal lesions (Speight et al., 2018; Aguirre-Urizar et al., 2021; Warnakulasuriya et al., 2021; Mohideen et al., 2025). Oral submucous fibrosis and oral lichen planus are included within the broader OPMD spectrum but should not be treated as biologically equivalent to classical plaque-like epithelial precursor lesions.

Early OSCC is considered the proximal invasive endpoint and a biologically informative comparator. Histologically, the transition from OPMD to OSCC is defined by invasion beyond the basement membrane. Direct human OPMD evidence supports altered epithelial differentiation, clonal field-risk features, transformation-linked metabolomic remodeling, and immune remodeling (Rosin et al., 2000; Schaaij-Visser et al., 2010; Farah et al., 2019; Weber et al., 2020; Wils et al., 2020; Zhang et al., 2024; Ferrari et al., 2025; Martins-Chaves et al., 2025). By contrast, wound-repair-like programs, epithelial-stromal interface architectures, and immune-exclusion-like patterns are mainly extrapolated from head and neck squamous cell carcinoma (HNSCC) or OSCC studies and should remain OSCC-derived inference (Puram et al., 2017; Ji et al., 2020; Pal et al., 2021; Arora et al., 2023).

### 2.2 Definition of the SMMI framework and candidate SMMI microdomains

The SMMI framework is an evidence-ranked conceptual and validation framework that integrates spatial organization, microbial signals, metabolic mediation, and immune remodeling within oral host tissue. A candidate SMMI microdomain is a pathology-defined local tissue region in which prespecified microbial and host features show quantitatively assessable spatial association through co-localization or adjacency. An integrated candidate SMMI microdomain is a higher-order candidate configuration in which multiple microbial and host layers converge within the same local region. These terms describe testable tissue configurations rather than fixed anatomical structures, established causal mechanisms, or validated therapeutic targets.

In this Review, spatial association is used as an umbrella term for co-localization and adjacency. Co-localization denotes signal overlap or assignment to the same pixel, spot, cell, segmented object, or other prespecified analytical unit at assay resolution. Spatial adjacency denotes the non-random proximity of prespecified microbial and host features within a pathology-defined tissue compartment, assessed using a prespecified distance or neighborhood rule appropriate to the biological hypothesis and assay resolution. Its quantitative implementation is detailed in Section 9.

Three principles guide the framework. Spatiality recognizes that risk may be unevenly distributed across a lesion. Coupling asks whether microbial, epithelial, metabolic, stromal, and immune alterations become locally coordinated rather than acting as independent lesion-level variables. Dynamism reflects repeated cycles of microbial exposure, epithelial injury, repair, barrier disruption, immune activation, immune tolerance, metabolic adaptation, and clonal selection.

Microbial signals are interpreted as candidate components of spatial host–microbe interaction. A bacterial, fungal, or viral signal near dysplastic epithelium may be contributory, permissive, reactive, passenger-like, contaminant-derived, or secondary to altered tissue architecture. Candidate SMMI microdomains therefore require pathology-anchored spatial registration, contamination-aware microbial validation, longitudinal sampling, functional perturbation, and outcome-linked testing.

### 2.3 Scope and evidence boundary

This Review focuses on oral microbe–host immune-metabolic spatial biology during the transition from high-risk OPMDs to early OSCC. Classical epithelial-risk alterations are discussed only when they intersect with barrier responses, microbial localization, metabolic remodeling, immune visibility, or candidate microdomain organization (Slaughter et al., 1953; Rosin et al., 2000; Farah et al., 2019). Broad screening technologies, standalone salivary biomarkers, advanced OSCC, post-treatment ecology, and immunotherapy-associated remodeling are outside the main scope unless they directly inform spatial host–microbe validation.

Direct human OPMD evidence is prioritized. OSCC evidence is used to define spatially testable hypotheses rather than direct premalignant mechanisms. Preclinical perturbation supports biological plausibility but does not replace human OPMD validation. **Box 1** defines the evidence hierarchy used throughout this Review to distinguish association-level evidence, spatial nomination, functional perturbation, and longitudinal clinical utility.

Box 1 **Evidence hierarchy for the oral microbe–host immune-metabolic spatial framework.**

• Level 1. Association-level evidence. Saliva, rinse, swab, scraping, tissue-homogenate, bulk omics, and single-marker studies can associate microbial, epithelial, metabolic, stromal, or immune features with OPMDs, dysplasia, OSCC, or transformation-related phenotypes. This level does not resolve spatial localization, temporal order, viability, activity, or causality.

• Level 2. Spatial association evidence. Spatial transcriptomics, multiplex imaging, microbial fluorescence *in situ* hybridization (FISH), RNAscope-like assays, spatial metabolomics, and registered serial-section mapping can test co-localization or adjacency and nominate candidate SMMI microdomains. This level does not establish temporal precedence, functional dependency, microbial viability, or causality.

• Level 3. Functional perturbation evidence. Co-culture systems, primary keratinocytes, air-liquid interface mucosa, organoids, organoid-immune-microbe systems, organ-on-chip platforms, 4-nitroquinoline 1-oxide (4NQO) models, and microbiome-modified animal models can test biological plausibility but do not by themselves establish natural human OPMD progression.

• Level 4. Longitudinal prediction and clinical utility. Retrospective-prospective formalin-fixed, paraffin-embedded (FFPE) cohorts, serially sampled OPMD cohorts, externally validated multimodal models, and outcome-linked studies can assess temporal precedence, incremental prediction, reproducibility, and clinical utility.

• Most current evidence supports individual SMMI components at Levels 1–3. Integrated candidate SMMI microdomains with longitudinal predictive value in human high-risk OPMDs remain a Level 4 validation goal.

## 3 Oral mucosa as a spatial host–microbe barrier ecosystem

### 3.1 Histopathology as the anchor for spatial interpretation

Histopathological grading of oral epithelial dysplasia remains indispensable because it captures architectural disturbance, cytological atypia, maturation abnormalities, and epithelial involvement (Kujan et al., 2007; Ranganathan and Kavitha, 2019; Wils et al., 2020). It also provides the tissue map on which spatial microbe–host hypotheses must be anchored. However, grading is constrained by sampling, interobserver variability, and intralesional heterogeneity, and it does not by itself resolve how epithelial abnormalities relate to microbial exposure, metabolic stress, stromal remodeling, or immune architecture. The SMMI framework should therefore complement, not replace, pathological assessment; any candidate SMMI microdomain must be interpreted as additional spatial context rather than a substitute for dysplasia grading.

### 3.2 Oral mucosa as a compartmental barrier ecosystem

Oral mucosa is a stratified microbe-exposed barrier that includes the surface biofilm and salivary interface, epithelial layers, basement membrane, epithelial–stromal interface, lamina propria, vasculature, lymphatics, stromal cells, and resident or recruited immune populations (Zijnge et al., 2010; Mark Welch et al., 2016; Moutsopoulos and Konkel, 2018; Pelaez-Prestel et al., 2021; Senel, 2021). Surface microbial products meet differentiated epithelium first, basal and suprabasal compartments maintain renewal and stress responses, and the interface and lamina propria organize immune access, tolerance, vascular signaling, and stromal remodeling. This compartmental architecture provides the anatomical basis for asking whether microbial, epithelial, metabolic, stromal, and immune features become locally coordinated within a lesion.

### 3.3 Candidate transformation-associated microdomains

Candidate transformation-associated microdomains are putative microregional configurations in which high-risk epithelial states coexist with microbial stimulation, metabolic stress, stromal remodeling, or altered immune surveillance. Human OPMD studies support altered epithelial maturation, field-risk changes, metabolomic remodeling, macrophage polarization, and indoleamine 2,3-dioxygenase 1 (IDO1)-positive macrophage-associated immunoregulation, while oral microbiome studies show context-dependent microbial shifts influenced by sampling source, lesion phenotype, site, periodontal status, oral hygiene, exposure history, antibiotics, and sequencing methods (Rosin et al., 2000; Schaaij-Visser et al., 2010; Hajishengallis and Lamont, 2012; Salter et al., 2014; Amer et al., 2017; Eisenhofer et al., 2019; Farah et al., 2019; Weber et al., 2020; Wils et al., 2020; Gopinath et al., 2021; Pietrobon et al., 2021; Herreros-Pomares et al., 2023; Wright et al., 2023; Spiljak et al., 2024; Zhang et al., 2024; Ferrari et al., 2025; Fierer et al., 2025; Galvin et al., 2025; Martins-Chaves et al., 2025). OSCC spatial studies remain useful comparators but should be treated as OSCC-derived inference rather than direct human OPMD proof (Ji et al., 2020; Arora et al., 2023). Section 3 therefore frames the anatomical rationale for candidate SMMI microdomains; detailed evidence classes and validation needs are summarized in **Table 1**.

**Table 1:** Evidence matrix for oral microbe–host SMMI layers in high-risk OPMDs and early OSCC.

SMMI layer	Representative evidence	Evidence class	SMMI relevance	Main limitation	Priority validation
Microbial dysbiosis/biofilm ecology	Context-dependent enrichment of anaerobic, periodontal, fungal, or polymicrobial signals in OPMD/OSCC studies.	Direct human OPMD evidence; OSCC-derived inference	Nominates candidate microbial signals at the biofilm-barrier interface.	Sampling source, periodontal status, contamination, and taxa variability.	Lesion-directed sampling with periodontal covariates and spatial validation.
Tissue-associated microbial localization	FISH, RNAscope-like assays, and registered host–microbe imaging can localize selected signals.	Methods paper; OSCC-derived inference	Tests whether microbial signals occur near host epithelial, immune, or metabolic regions.	Localization does not prove viability, activity, or causality.	Orthogonal *in situ* validation with controls and host-context co-staining.
Epithelial barrier-response states	Impaired differentiation, basal-like expansion, clonal field-risk and stress-response programs.	Direct human OPMD evidence; OSCC-derived inference	Defines the susceptible host epithelial substrate for candidate microdomains.	Epithelial risk states alone are not microbe–host niches.	Co-register epithelial states with microbial localization and outcomes.
Microbe-linked metabolic pressures	Lipid remodeling, oxidative stress, lactate/hypoxia inference, tryptophan-kynurenine biology.	Direct human OPMD association; broader cancer biology	Provides biochemical mediation between microbes, epithelium, macrophages and T cells.	Bulk metabolomics cannot assign source or spatial gradients.	Spatial metabolomics registered to microbial and immune imaging.
Macrophage-centered immune remodeling	indoleamine 2,3-dioxygenase 1 (IDO1)-positive macrophages and macrophage polarization associated with leukoplakia transformation.	Direct human OPMD evidence	Nominates a candidate immunometabolic host-response layer.	Does not prove microbial induction, kynurenine accumulation or T-cell suppression.	Map IDO1+ macrophages with microbial signals, metabolites and T-cell distance.
Integrated candidate SMMI microdomain	Proposed convergence of microbial signals, epithelial states, metabolic stress and immune remodeling.	Speculative but testable hypothesis	Provides a framework for spatial ecology and risk-stratification studies.	Integrated candidate SMMI microdomains have not been validated in human OPMDs.	Longitudinal OPMD cohorts, perturbation testing and external validation.

FISH, fluorescence *in situ* hybridization; IDO1, indoleamine 2,3-dioxygenase 1; OPMD, oral potentially malignant disorder; OSCC, oral squamous cell carcinoma; SMMI, spatial–microbial–metabolic–immune.

## 4 Epithelial host substrates relevant to candidate SMMI microdomains

High-risk epithelial states provide the host substrate on which microbe-associated SMMI microdomains may be nominated. In a microbe-exposed oral barrier, epithelial alterations should be interpreted not only as lesion-intrinsic oncogenic changes, but also as barrier-response states that may shape, amplify, restrain, or reflect microbial ecology, inflammatory tone, metabolic stress, stromal remodeling, and immune visibility. However, epithelial alterations alone do not define an oral microbe–host immune-metabolic niche. Their SMMI relevance depends on whether they are spatially registered with microbial localization, host microbial-response programs, metabolic readouts, immune-cell positioning, and longitudinal outcomes.

### 4.1 Basal-like expansion and impaired differentiation

Oral epithelial dysplasia and OSCC are characterized by disturbed stratified differentiation. Basal/progenitor-associated programs marked by KRT5, KRT14, TP63, and ITGA6 may expand, whereas differentiation-associated markers such as KRT4, KRT13, and cornulin may decrease (Schaaij-Visser et al., 2010; Wils et al., 2020). In human OPMDs, impaired epithelial maturation is a progression-relevant host state, but it should not be interpreted as a microbe-associated niche unless linked to microbial and immune-metabolic spatial context.

From a microbe–host barrier perspective, impaired differentiation may alter keratinization, epithelial permeability, antimicrobial responses, surface adhesion, and biofilm retention. Conversely, microbial stimulation, epithelial injury, and inflammatory repair may reinforce abnormal differentiation in susceptible epithelial fields. The SMMI question is therefore whether differentiation-impaired regions spatially align with biofilm-barrier interfaces, microbial-response programs, metabolic stress, stromal remodeling, or immune restriction before invasion.

### 4.2 Clonal field-risk as host susceptibility

Field cancerization emphasizes that visible OPMDs may arise within broader mucosal fields shaped by carcinogen exposure, clonal expansion, and genetically altered epithelium (Slaughter et al., 1953). TP53, CDKN2A, copy-number changes, chromosomal instability, and loss of heterozygosity are risk-related epithelial layers in oral leukoplakia and oral cavity carcinogenesis (Rosin et al., 2000; Farah et al., 2019).

Within the SMMI framework, these alterations define host epithelial susceptibility rather than a microbe–host niche itself. A genetically altered field may respond differently to microbial products, inflammatory mediators, oxidative stress, barrier injury, or immune surveillance. However, clonal risk does not demonstrate microbial, metabolic, or immune coupling unless it is spatially registered with microbial localization, epithelial barrier-response programs, metabolic stress, stromal remodeling, or immune-cell positioning.

### 4.3 Wound-repair-like plasticity

Partial EMT-like programs in HNSCC involve hybrid epithelial states related to extracellular matrix interaction, altered adhesion, inflammatory signaling, collective migration, and wound repair (Puram et al., 2017; Pal et al., 2021). For high-risk OPMDs, this evidence should remain OSCC-derived inference. Before basement membrane breach, wound-repair-like or partial EMT-like plasticity should not be interpreted as invasion or as direct evidence of an established premalignant SMMI framework.

A more appropriate barrier-centered formulation is that subsets of dysplastic epithelium may activate repair-associated host-response programs involving altered adhesion, matrix interaction, inflammatory responsiveness, antimicrobial signaling, and stress adaptation. These programs may be shaped by epithelial injury, microbial stimulation, hypoxia, stromal remodeling, or macrophage-derived mediators. Their SMMI relevance requires spatial evidence that repair-like epithelial states occur near microbial signals, metabolic pressures, or immune-restricted stromal regions in human OPMDs.

### 4.4 DNA damage, oxidative stress and immune visibility

Dysplastic epithelium is exposed to carcinogens, chronic inflammation, reactive oxygen and nitrogen species, hyperproliferation, and potentially microbe-linked biochemical stress. Oral microbial communities may contribute through inflammatory stimulation, acetaldehyde-related metabolism, redox-active products, proteolytic activity, and polymicrobial biofilm organization, but these contributions are context-dependent and are supported mainly by broader oral carcinogenesis and preclinical evidence (Uitto et al., 2005; Gallimidi et al., 2015; Perera et al., 2016; Sztukowska et al., 2016; Geng et al., 2017; Lee et al., 2017).

Epithelial immune visibility is another host substrate relevant to SMMI research. Dysplastic epithelial cells may become more recognizable through DNA damage, stress signaling, altered differentiation, or microbial-response pathways, but they may also reduce immune engagement through impaired antigen presentation, altered interferon responsiveness, or local immunosuppressive circuits. Direct epithelial-specific evidence for antigen-presentation deficits in OPMDs remains limited (Yoshihama et al., 2016; DhatChinamoorthy et al., 2021; Wickenhauser et al., 2021; Attaran et al., 2022). The key spatial question is whether epithelial regions with clonal risk, impaired differentiation, barrier disruption, or microbial-response programs show altered relationships with CD8+ T cells, regulatory T cells (Tregs), macrophage states, MHC-I/antigen-presentation markers, and tryptophan-kynurenine-related signals.

### 4.5 Evidence boundary

Epithelial host substrates are supported by meaningful direct human OPMD evidence for impaired differentiation, dysplasia-associated risk states, and selected field-risk alterations. OSCC-derived evidence also provides useful hypotheses regarding wound-repair-like plasticity and altered epithelial immune visibility. However, basal-like expansion, differentiation failure, clonal alteration, wound-repair-like plasticity, oxidative stress, or altered immune visibility should be treated as host substrates rather than SMMI microdomains by themselves. Their relevance to the spatial oral microbe–host immune-metabolic framework depends on co-registration with microbial localization, metabolic readouts, immune-cell positioning, and outcome-linked validation.

## 5 Microbial ecology and host-response amplification

Microbial ecology is the microbiological anchor of the SMMI framework. Oral microbiome studies associate OPMDs and OSCC with altered bacterial, fungal, and polymicrobial communities, but no single microbial signature consistently defines malignant transformation. The relevant question is whether lesion-associated communities, products, or host microbial-response programs are positioned to influence, reflect, or amplify epithelial barrier disruption, metabolic stress, stromal remodeling, and immune regulation.

### 5.1 Dysbiosis, biofilm ecology and sampling source

Oral microbial ecology is shaped by salivary flow, mucosal structure, dental plaque, periodontal status, oxygen tension, diet, tobacco, alcohol, areca exposure, oral hygiene, antimicrobials, and anatomical site (Zijnge et al., 2010; Hajishengallis and Lamont, 2012; Mark Welch et al., 2016; Lamont et al., 2018; Radaic and Kapila, 2021). OPMD and OSCC studies report context-dependent anaerobic, inflammation-associated, periodontal, fungal, or polymicrobial signals, but taxa vary across cohorts and should not be treated as a reproducible universal OPMD signature (Amer et al., 2017; Shukla et al., 2019; Nejman et al., 2020; Zhang et al., 2020; Pietrobon et al., 2021; Galeano Niño et al., 2022; Herreros-Pomares et al., 2023; Wright et al., 2023; Cai et al., 2024; Galvin et al., 2025). Surface biofilms may alter oxygen tension, pH, nutrient availability, proteolytic activity, and inflammatory tone, while dysplastic or keratin-altered surfaces may themselves favor selected organisms.

Sampling source determines interpretation. Saliva and oral rinse are scalable but cannot establish lesion-localized contact. Swabs and scrapings capture surface communities but are affected by keratinization, saliva, plaque, periodontal status, and adjacent fields. Tissue sequencing adds histopathological context but is vulnerable to low biomass and contamination during collection, processing, extraction, and analysis (Salter et al., 2014; Eisenhofer et al., 2019; Gopinath et al., 2021; Spiljak et al., 2024; Fierer et al., 2025). FISH, RNAscope-like assays, and related *in situ* methods can localize selected organisms or transcripts, but probe specificity and section contamination require validation (Amann et al., 1995; Valm et al., 2011; Wang et al., 2012; Cao et al., 2023).

Microbial DNA should not be equated with viability, activity, or causal contribution. A detected organism may act as a driver, passenger, risk marker, or ecological amplifier depending on lesion and periodontal context (Hajishengallis and Lamont, 2012; Galvin et al., 2025). Detailed sampling, localization, and evidentiary boundaries are summarized in **Table 2** and operationalized in Section 9 and **Box 2**.

**Table 2:** Microbial sampling sources and interpretive boundaries for oral microbe–host SMMI research.

Sampling or detection approach	What it can support	What it cannot prove/key caveats
Saliva or oral rinse	Patient-level microbial dysbiosis; scalable profiling.	Lesion-localized microbial contact or spatial compartment localization.
Lesion swab or scraping	Surface lesion-associated community structure.	Microbial invasion, viability, causality, or stromal localization.
Tissue biopsy sequencing	Tissue-associated taxa paired with histopathology.	Exact localization without imaging; contamination-free signal without controls.
16S rRNA sequencing	Broad taxonomic profiling.	Species-level certainty, microbial function, viability, or spatial distribution.
Shotgun metagenomics	Improved species and functional inference.	Spatial localization or robust interpretation in low-biomass tissue without controls.
Microbial FISH/RNAscope-like assays	Spatial localization of selected organisms or transcripts.	Causality, temporal precedence, community-wide function, or viability.
Registered host–microbe imaging	Candidate microdomain nomination in host tissue context.	Functional contribution without perturbation and outcome-linked validation.

Box 2 **Minimum reporting standards for spatial oral microbe–host SMMI studies.**

• Define lesion type, anatomical site, phenotype, dysplasia grade, keratinization, inflammation, and outcome where available.

• Report sampling source and workflow, including handling, fixation, embedding, sectioning, extraction, processing, and batch structure.

• Record relevant exposure and oral ecological covariates, including periodontal status, plaque burden, tooth proximity, tobacco, alcohol and areca exposure, oral hygiene, recent dental treatment, and antimicrobial exposure where feasible.

• Prespecify the pathology-defined compartment, distance or neighborhood scale, spatial metric, null model, threshold rationale, assay resolution, and registration uncertainty used to define spatial adjacency.

• Distinguish co-localization at assay resolution from adjacency within a prespecified neighborhood, and do not treat descriptive spatial alignment as quantitative evidence.

• Define abnormality against an explicit within-lesion, patient-matched, or external healthy comparator, and treat adjacent normal-appearing mucosa as patient-matched reference tissue when field effects may persist.

• Include microbial negative controls, blank extraction controls, no-template controls, reagent controls, probe controls, and section-order controls.

• Distinguish saliva-, swab-, tissue sequencing-, and spatial detection-based evidence.

• Validate key microbial signals by orthogonal *in situ* methods when claiming localization.

• Distinguish microbial DNA signal from viability, activity, host-response co-localization, and causality.

• Register microbial, epithelial, metabolic, immune, stromal, and histopathological layers on same or adjacent sections.

• Report spatial resolution, segmentation, cell-state annotation, metabolite annotation confidence, and uncertainty.

• Use causal language only when supported by temporal, perturbation, and outcome-linked evidence.

## 6 Microbe-linked metabolic pressures and biochemical mediation

Metabolism provides a biochemical interface through which microbial ecology, epithelial barrier stress, stromal remodeling, macrophage states, and T-cell function may connect within candidate SMMI microdomains. In oral malignant transformation, metabolic remodeling is most useful when framed as a candidate mediation layer linking microbe-associated irritation, epithelial stress adaptation, inflammation, and immune regulation.

### 6.1 Metabolism as microbe–host crosstalk

Most OPMD metabolomic studies use saliva, serum, tissue homogenates, or bulk platforms. These approaches can identify transformation- or dysplasia-associated metabolic differences but usually cannot assign metabolite source or determine whether metabolic signals localize near microbial communities, dysplastic epithelium, macrophage-rich regions, or T-cell-restricted compartments (Wei et al., 2011; Sridharan et al., 2019; Ferrari et al., 2025; Martins-Chaves et al., 2025). A metabolite associated with high-risk disease may reflect epithelial stress, microbial metabolism, immune activity, stromal remodeling, periodontal inflammation, diet, or mixed sources.

SMMI research should therefore ask whether candidate metabolites are enriched near microbial signals or biofilm-barrier interfaces; whether they align with epithelial stress or impaired differentiation; and whether they occur near macrophage-centered immunoregulation, altered CD8+ T-cell positioning, or reduced epithelial immune visibility.

### 6.2 Lipid remodeling and biochemical stress

Human OPMD metabolomic studies suggest that malignant-transformed and non-transformed oral leukoplakias may differ in lipid-related features, including phospholipids, oxidized lipids, cholesteryl esters, and fatty acids (Martins-Chaves et al., 2025). Salivary metabolomic differences between leukoplakia with and without dysplasia also support the possibility that metabolic profiles reflect epithelial-risk states (Ferrari et al., 2025).

For SMMI research, lipid remodeling should be interpreted as a candidate host-response and membrane-stress layer rather than a standalone biomarker signature. Lipid species may reflect epithelial proliferation, membrane turnover, oxidative stress, inflammatory mediators, microbial community effects, immune-cell activity, or stromal remodeling. Current evidence supports prioritization of lipid remodeling for spatial validation within host–microbe tissue context.

### 6.3 Acetaldehyde, oxidative stress, lactate and kynurenine-related axes

Several biochemical pressures may connect oral microbial ecology with host responses. Acetaldehyde-related stress may link microbial metabolism, alcohol exposure, fungal or bacterial activity, epithelial genotoxic stress, and mucosal irritation. Reactive oxygen and nitrogen species may arise from inflammation, epithelial stress, immune cells, and microbial stimulation. Fermentation-related products and short-chain-fatty-acid-related signals may influence epithelial differentiation, barrier function, inflammatory signaling, or immune tone depending on concentration and tissue context.

Lactate and hypoxia-related features are plausible immunometabolic layers because they connect epithelial stress adaptation, glycolysis, stromal remodeling, and immune function, mainly based on broader cancer immunometabolism evidence (Brand et al., 2016). However, broader cancer evidence should not be used to claim that lactate gradients drive immune restriction in OPMDs. Similarly, the tryptophan-kynurenine pathway links metabolism and immune regulation (Platten et al., 2019), and IDO1-positive macrophages associated with oral leukoplakia transformation nominate an important immunometabolic axis (Zhang et al., 2024). Local metabolite accumulation, microbial induction, T-cell suppression, temporal contribution, and causal relevance should be tested through spatial registration, perturbation, and outcome-linked studies.

### 6.4 Spatial metabolomics and evidence boundary

Spatial metabolomics, including matrix-assisted laser desorption/ionization mass spectrometry imaging, can map lipid species, oxidative-stress products, lactate-associated signals, acetaldehyde-related features, or kynurenine-pathway metabolites in tissue sections (Buchberger et al., 2018). Its value lies in registration with histopathology, microbial localization, epithelial-state mapping, and immune imaging.

Current evidence supports metabolic remodeling as a candidate mediation layer, but the metabolome has weaker direct spatial evidence in human high-risk OPMDs than epithelial and immune layers. Lactate, kynurenine, IDO1, and lipid remodeling remain candidate axes requiring co-registration with microbial localization, epithelial host-response states, macrophage and T-cell positioning, perturbation evidence, and longitudinal outcomes.

## 7 Microbiome-associated immune remodeling and host-response restriction

The oral mucosa maintains immune surveillance while encountering commensal microorganisms, pathobionts, dietary antigens, mechanical injury, plaque, and carcinogen-associated stress. In high-risk OPMDs, immune remodeling is best framed as mucosal barrier immunology in a microbe-exposed tissue environment, where microbial stimulation, epithelial injury, metabolic stress, stromal remodeling, and clonal vulnerability may shape immune-cell recruitment, positioning, and function.

### 7.1 Immune surveillance and tolerance

Normal oral mucosa balances immune defense and tolerance. Resident and recruited immune populations are distributed across the epithelium, epithelial-stromal interface, and lamina propria (Moutsopoulos and Konkel, 2018; Pelaez-Prestel et al., 2021; Senel, 2021). Immune-cell position may be as relevant as abundance. A CD8+ T cell distant from dysplastic epithelium differs biologically from one in direct epithelial proximity, and macrophages clustered near a disrupted biofilm-barrier interface may differ from diffusely distributed lamina propria macrophages.

A dysplastic region does not evolve in an immunological vacuum. It is embedded within a microbe-exposed environment shaped by local tolerance, microbial stimulation, inflammatory memory, epithelial barrier integrity, stromal organization, and metabolic gradients.

### 7.2 Host-response restriction and macrophage-centered immunoregulation

Chronic inflammation is associated with oral carcinogenesis, but inflammatory infiltration alone does not define high risk. The more relevant question is whether microbial stimulation, epithelial injury, oxidative stress, and repair responses shift local immunity toward host-response restriction. This may involve macrophage-centered immunoregulation, Treg enrichment, altered antigen presentation, checkpoint-associated signaling, stromal barriers, metabolic suppression, or inefficient CD8+ T-cell positioning.

Cancer immunoediting and broader tumor-immune microenvironment concepts are useful theoretical backgrounds but should be applied cautiously to premalignant mucosa (Schreiber et al., 2011; Binnewies et al., 2018). In OPMDs, terms such as immune-editing-like remodeling, early host-response restriction, or altered epithelial immune visibility are more appropriate than fully developed tumor immune escape.

The strongest direct human OPMD-related evidence comes from oral leukoplakia studies identifying IDO1-positive macrophages as a candidate immunosuppressive population associated with malignant transformation (Zhang et al., 2024). Independent work also links macrophage polarization with leukoplakia transformation (Weber et al., 2020). These findings connect premalignant progression to macrophage state and immunoregulatory signaling, while microbial induction, local kynurenine accumulation, T-cell suppression, and temporal precedence remain to be tested.

### 7.3 T-cell positioning and epithelial immune visibility

In OPMDs, a premalignant-appropriate formulation is compartmentalized host-response restriction rather than established immune exclusion. Immune cells may be present but inefficiently positioned, metabolically constrained, or functionally limited relative to dysplastic epithelium. CD8+ T cells may remain in the lamina propria without engaging epithelial risk domains; macrophages or Tregs may cluster near epithelial-stromal interfaces; and stromal remodeling may restrict immune access.

Bulk RNA, single-marker immunohistochemistry, or whole-section quantification cannot determine these spatial relationships. Spatial imaging or spatial transcriptomics is needed to assess CD8+ T-cell distance to dysplastic epithelium, macrophage–T-cell proximity, FOXP3+ Treg enrichment, epithelial HLA class I or β2-microglobulin (B2M) expression, and co-localization with microbial or metabolic signals.

Epithelial immune visibility is underdeveloped in OPMD research. High-risk epithelial clones may accumulate DNA damage, stress signals, altered differentiation, or microbial-response pathways that increase recognition, but may also reduce immune engagement through impaired interferon responsiveness, antigen processing, MHC class I presentation, or checkpoint-associated signaling. OSCC data on HLA class I and antigen-processing machinery remain hypothesis-generating for premalignant disease (Yoshihama et al., 2016; DhatChinamoorthy et al., 2021; Wickenhauser et al., 2021; Attaran et al., 2022).

### 7.4 Checkpoint markers, preclinical models and evidence boundary

Checkpoint-related markers such as PD-1, PD-L1, LAG3, and TIGIT are relevant to oral carcinogenesis, but their interpretation in OPMDs should remain conservative (Yagyuu et al., 2017; Dave et al., 2020). They are best treated as candidate immune readouts that require spatial context, epithelial-state registration, microbial or metabolic linkage, and outcome validation.

Preclinical models, including 4NQO systems, can test whether macrophage states, T-cell positioning, antigen-presentation programs, microbial exposure, or metabolic stress contribute to lesion progression-related phenotypes (Gallimidi et al., 2015; Sellaeg et al., 2024). However, carcinogen-induced lesions, murine immunity, microbiota composition, exposure dose, anatomy, and kinetics differ from human OPMDs. Such models provide functional plausibility and temporal support, not direct proof of natural human premalignant progression.

Current immune evidence supports the plausibility of immune remodeling during oral malignant transformation, especially macrophage-centered immunoregulation. However, immune-editing-like remodeling, host-response restriction, altered T-cell positioning, epithelial immune visibility, and checkpoint-associated markers remain spatially testable layers rather than established causal mechanisms. Their SMMI relevance requires co-registration with microbial localization, epithelial states, metabolic readouts, longitudinal outcomes, and perturbation evidence.

## 8 Candidate oral microbe–host SMMI microdomains: a working spatial framework

**Figure 1** summarizes the proposed spatial oral microbe–host framework and the candidate SMMI microdomains that may be nominated during the transition from high-risk OPMDs to early OSCC. The framework guides spatial hypothesis generation rather than defining a fixed anatomical entity or established causal mechanism. It highlights four interrelated host–microbe response layers: the surface biofilm-barrier microdomain, host epithelial barrier-response microdomain, microbe-linked metabolic mediation microdomain, and immune-restricted stromal microdomain.

**Figure 1:**
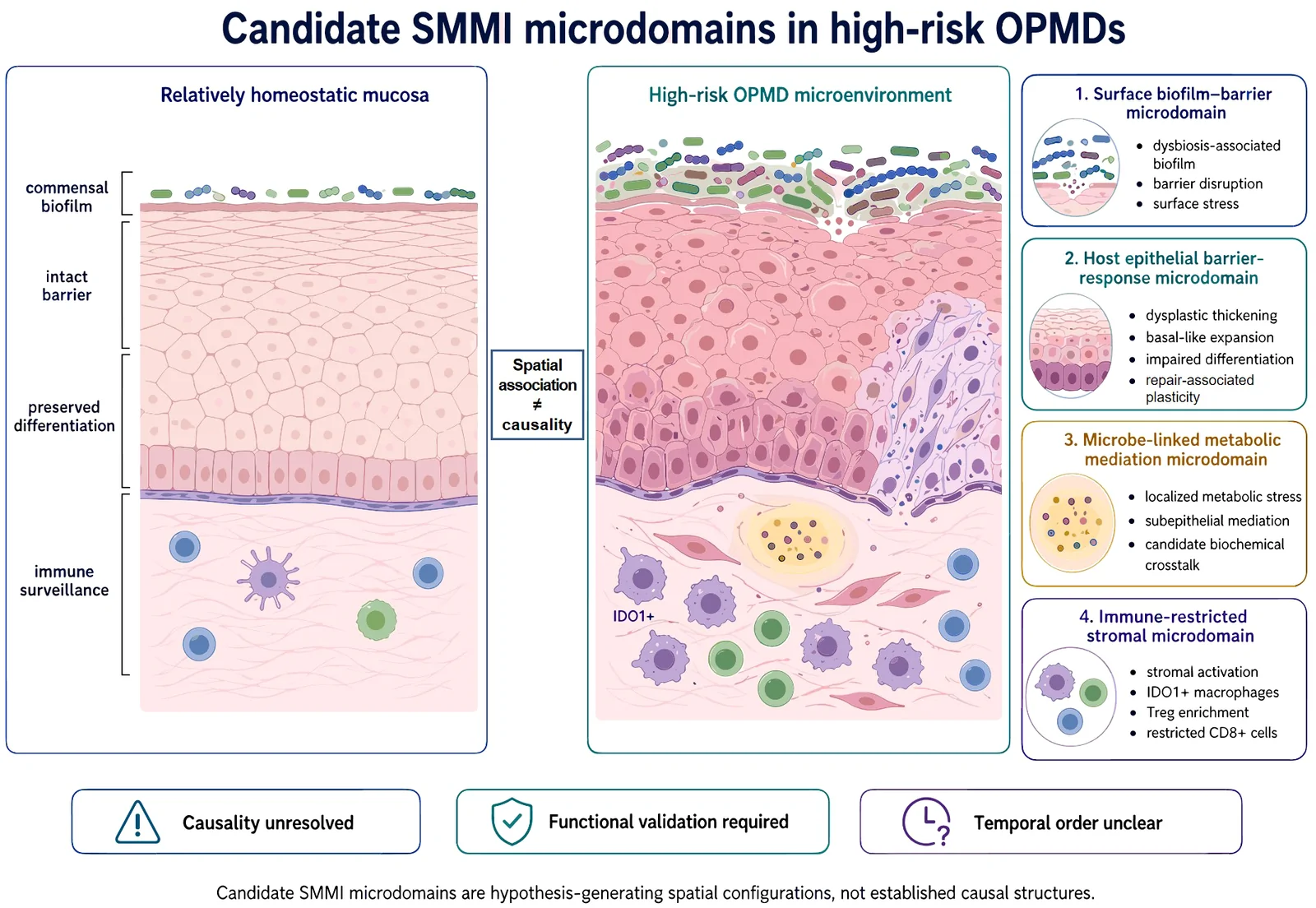
Spatial oral microbe-host ecology in high-risk OPMDs: candidate SMMI microdomains. This framework depicts candidate SMMI microdomains that may be nominated during progression from high-risk oral potentially malignant disorders (OPMDs) toward early oral squamous cell carcinoma. The surface biofilm-barrier microdomain is shown as the immediate interface between microbial communities and the mucosal barrier, while host epithelial barrier-response, microbe-linked metabolic mediation, and immune-restricted stromal microdomains represent parallel host-response layers that may become spatially associated with microbial signals. These microdomains are proposed as measurable, lesion-restricted configurations rather than proven mechanisms. The figure therefore emphasizes that spatial association is not equivalent to causality: temporal order remains unclear, causality is unresolved, and functional validation is required. Repair-associated plasticity is included to capture epithelial and stromal adaptation that may accompany chronic microbial, metabolic, or inflammatory stress. The framework is intended to guide spatially registered sampling, orthogonal microbial localization, and hypothesis-driven validation in high-risk OPMD research.

### 8.1 Surface biofilm-barrier microdomain

The surface biofilm-barrier microdomain is the microbiological anchor of the framework because it directly connects oral microbial ecology with epithelial host responses. It refers to a mucosal interface where biofilm ecology, altered differentiation, barrier disruption, and inflammatory signaling occur in proximity. Surface-associated microbes may act as context-dependent ecological amplifiers by sustaining irritation, modifying oxygen tension or nutrients, stimulating pattern-recognition pathways, affecting antimicrobial responses, and reinforcing repair programs. Conversely, dysplastic or keratin-altered surfaces may favor selected anaerobic, pathobiont, fungal, or polymicrobial communities.

Candidate readouts include the broad bacterial probe EUB338, targeted FISH or RNAscope-like detection of *Fusobacterium*, *Porphyromonas, Prevotella*, or other nominated taxa, *Candida* detection where justified, reduced KRT4/KRT13/cornulin, barrier-response markers, IL-1β, IL-6, IL-8, Toll-like receptor (TLR)/nuclear factor-κB (NF-κB)-response programs, and epithelial stress markers. These are candidate readouts, not validated predictors. Validation requires lesion-directed sampling, contamination-aware microbial localization, registered host–microbe imaging, serial-section microbial detection with epithelial markers, multiplex imaging for inflammatory responses, and spatial transcriptomics for host microbial-response programs. Microbial presence at the surface does not prove viability, activity, temporal precedence, or causality.

### 8.2 Host epithelial barrier-response microdomain

The host epithelial barrier-response microdomain includes spatially restricted epithelial regions enriched for impaired differentiation, basal-like expansion, clonal field-risk features, stress tolerance, wound-repair-like plasticity, or altered immune visibility. Dysplastic epithelium remains central, but its SMMI relevance depends on whether epithelial risk states are spatially connected to microbial signals, metabolic pressures, stromal remodeling, or immune-cell positioning.

Candidate readouts include basal/progenitor markers, differentiation markers, TP53/CDKN2A-related alterations, loss of heterozygosity (LOH) and copy-number variation (CNV) indicators, DNA-damage or oxidative-stress markers, and HLA class I, β2-microglobulin (B2M), TAP1/2, NLRC5, or interferon-response markers. These features identify host epithelial vulnerability but do not demonstrate microbe–host coupling unless spatially registered with microbial, metabolic, stromal, or immune features.

### 8.3 Microbe-linked metabolic mediation microdomain

The microbe-linked metabolic mediation microdomain refers to regions in which metabolic readouts may connect microbial ecology, epithelial barrier stress, stromal remodeling, macrophage states, and T-cell positioning. Candidate readouts include lipid remodeling, oxidized lipids, acetaldehyde-related stress, oxidative-stress products, hypoxia/glycolysis-related signals, lactate-related readouts, and tryptophan-kynurenine-pathway metabolites.

Spatial metabolomics and adjacent-section registration can test whether these readouts are enriched near biofilm-barrier regions, epithelial barrier-response domains, IDO1-positive macrophages, or CD8+ T-cell-restricted stromal areas. Source attribution, immune relevance, temporal order, and functional dependency require further validation.

### 8.4 Immune-restricted stromal microdomain

The immune-restricted stromal microdomain refers to stromal or epithelial-stromal regions where immune cells are present but inefficiently positioned, metabolically constrained, or functionally limited relative to epithelial risk domains. This concept is narrower than established tumor immune exclusion.

Candidate readouts include IDO1-positive macrophages, CD68/CD163/CD206 macrophage markers, CD8+ T-cell distance to dysplastic epithelium, FOXP3+ regulatory T cells (Tregs), PD-1/PD-L1, HLA class I, β2-microglobulin (B2M), TAP1/2, NLRC5, and kynurenine-, lactate-, or hypoxia-related markers. These should be analyzed as spatial relationships rather than isolated abundance. Immune-cell abundance or checkpoint expression alone does not demonstrate immune restriction or readiness for immune-targeted intervention.

### 8.5 Integrated microdomains as testable hypotheses

The four microdomains may coexist, overlap, or vary across lesion regions. Their practical value lies in converting oral microbial ecology and host-response concepts into measurable spatial readouts. A high-value SMMI study would test whether these readouts are spatially registered in histologically defined OPMD regions, differ between transforming and non-transforming lesions, add predictive value beyond dysplasia grade and clinical phenotype, and can be tested in perturbation systems. **Table 3** summarizes candidate oral microbe–host SMMI microdomains, representative validation readouts, suitable assays, and evidence boundaries.

**Table 3:** Candidate oral microbe–host SMMI microdomains and validation readouts.

Candidate microdomain	Host-response layer	Candidate readouts	Validation assays	Evidence boundary
Surface biofilm-barrier	Direct oral microbial ecology and epithelial barrier response.	EUB338, targeted microbial fluorescence *in situ* hybridization (FISH)/RNAscope, *Candida* where justified, KRT4/KRT13/cornulin, IL-1β/IL-6/IL-8, TLR/NF-κB.	Lesion-directed sampling, contamination-aware localization, registered host–microbe imaging.	Microbial localization does not prove viability, activity or causality.
Host epithelial barrier-response	Susceptible epithelial substrate exposed to microbial and inflammatory pressures.	KRT5/KRT14/TP63/ITGA6, KRT4/KRT13/cornulin, TP53/CDKN2A, loss of heterozygosity (LOH)/copy-number variation (CNV), HLA class I/β2-microglobulin (B2M)/TAP/NLRC5.	Spatial histopathology, multiplex epithelial markers, microdissection, spatial transcriptomics.	Epithelial risk states alone do not define a microbe–host niche.
Microbe-linked metabolic mediation	Biochemical mediation between microbes, epithelium, macrophages and T cells.	Lipids, oxidized lipids, acetaldehyde-related stress, lactate/hypoxia, kynurenine-pathway metabolites.	Spatial metabolomics registered to microbial and immune imaging.	Metabolic enrichment does not prove microbial source or immune suppression.
Immune-restricted stromal	Spatial host-response restriction near epithelial-stromal interfaces.	IDO1+ macrophages, CD8+ T-cell distance, FOXP3+ Tregs, PD-1/PD-L1, HLA class I/β2-microglobulin (B2M).	Multiplex immune imaging, spatial neighborhoods, macrophage-T-cell proximity.	Immune abundance or checkpoint expression alone does not prove immune escape.

B2M, β2-microglobulin; CNV, copy-number variation; FISH, fluorescence *in situ* hybridization; IDO1, indoleamine 2,3-dioxygenase 1; LOH, loss of heterozygosity; NF-κB, nuclear factor-κB; OPMD, oral potentially malignant disorder; SMMI, spatial–microbial–metabolic–immune; TLR, Toll-like receptor; Tregs, regulatory T cells.

Integrated candidate SMMI microdomains remain working hypotheses. The evidence is strongest for individual components, including microbial dysbiosis, epithelial host-response states, transformation-linked metabolic remodeling, and macrophage-centered immune changes. The decisive next step is to determine whether these layers are spatially coupled, temporally related to transformation, functionally linked, reproducible, and clinically informative.

## 9 A modular validation roadmap for candidate SMMI microdomains

A practical SMMI validation strategy should be modular and hypothesis-driven rather than technology-comprehensive. No single study is expected to combine every localization, spatial-omics, multiplex-imaging, longitudinal, and perturbation platform. Validation should instead proceed from a minimum pathology-anchored core to hypothesis-selected spatial modules, targeted functional testing, and longitudinal or clinical validation when justified by the preceding evidence. These study-design tiers are distinct from the evidence levels in **Box 1**, and histopathology remains the anchor throughout.

### 9.1 Tier 1 — minimum pathology-anchored core

Tier 1 is the minimum required core for nominating a candidate SMMI microdomain. It includes pathology-anchored lesion sampling, an explicit comparator, contamination-aware microbial controls, orthogonal *in situ* microbial localization, at least one hypothesis-relevant host readout, and an assay-appropriate spatial association rule. Comparators should be prespecified as within-lesion references, patient-matched reference mucosa, or external healthy controls matched or adjusted, where feasible, for anatomical site, exposure background, periodontal context, sampling, and processing. Adjacent normal-appearing mucosa should be treated as patient-matched reference tissue rather than an unequivocally healthy baseline because field effects may persist (Slaughter et al., 1953). Detailed sampling and reporting requirements are summarized in **Box 2**.

Co-localization and adjacency are related but distinct forms of spatial association. Co-localization denotes signal overlap or assignment to the same prespecified analytical unit at assay resolution, whereas adjacency denotes non-random proximity within a prespecified distance or neighborhood. Studies should define the pathology-based compartment and distance or neighborhood rule before analysis and report assay resolution, registration uncertainty, the selected spatial metric, the null model or comparator, and the threshold rationale. Metrics may include nearest-neighbor distance, distance to the epithelial boundary, fixed-radius enrichment, adjacent-spot or graph-based neighborhoods, or spatial cross-correlation; numerical cutoffs should be biologically and technically justified and, where feasible, tested for sensitivity to alternative thresholds (Palla et al., 2022). No universal micrometre cutoff is appropriate across microbial FISH, multiplex imaging, spatial transcriptomics, and spatial metabolomics (Stahl et al., 2016; Longo et al., 2021; Marx, 2021). Localization alone does not establish microbial viability, activity, temporal precedence, or causality (Amann et al., 1995; Valm et al., 2011; Wang et al., 2012; Salter et al., 2014; Eisenhofer et al., 2019; Cao et al., 2023; Fierer et al., 2025). Without a contamination-controlled and orthogonally confirmed localized microbial signal, a study should not automatically escalate to expensive integrated spatial profiling.

### 9.2 Tier 2 — hypothesis-selected spatial modules

Tier 2 is optional. One or more epithelial, immune, metabolic, or stromal/interface modules may be selected according to the biological hypothesis, specimen availability, assay resolution, and Tier 1 findings; all modules are not required. The epithelial module evaluates whether microbial signals align with differentiation, barrier-response, DNA-damage, or immune-visibility states. The immune module evaluates cell positioning and neighborhood structure; multiplex imaging may be selected when these are the primary questions, but abundance alone does not establish functional restriction. The metabolic module evaluates spatially localized metabolites when metabolic mediation is central to the hypothesis, while recognizing the difficulty of attributing metabolites to epithelial, immune, stromal, microbial, salivary, dietary, periodontal, or necrotic sources. The stromal/interface module evaluates whether basement-membrane, lamina-propria, vascular, or stromal remodeling organizes local host responses. **Table 3** summarizes representative readouts and assays without requiring simultaneous use (Angelo et al., 2014; Giesen et al., 2014; Buchberger et al., 2018; Goltsev et al., 2018; Lin et al., 2018). If a selected module does not reproduce a prespecified spatial association, the result should remain component-level evidence rather than trigger automatic functional escalation.

### 9.3 Tier 3 — targeted functional testing

Tier 3 is conditional and should test a specific relationship nominated by reproducible human spatial observations. The perturbation model should be matched to that relationship rather than applied as a broad platform screen. Options include microbe–epithelium co-culture, microbial-product or metabolite exposure, organotypic or air–liquid interface mucosa, organoids, organ-on-chip systems, and appropriate animal models, including 4NQO models (Bhatia and Ingber, 2014; Gallimidi et al., 2015; Drost and Clevers, 2018; Rahimi et al., 2018; Driehuis et al., 2019; Sellaeg et al., 2024). These systems do not provide equivalent evidence. Functional effects without concordant human OPMD spatial evidence support biological plausibility, not validation of a human candidate SMMI microdomain.

### 9.4 Tier 4 — longitudinal and clinical validation

Tier 4 is required for clinical-utility claims. Longitudinal and outcome-linked studies should test temporal precedence, transformation association, differences between transforming and non-transforming lesions, external reproducibility, and incremental prediction beyond dysplasia grade and established clinicopathological factors (Mahmood et al., 2023; Wright et al., 2023; Shephard et al., 2024). Retrospective–prospective FFPE cohorts can support validation but are not equivalent to serial longitudinal sampling. Predictive models require external validation and calibration. A feature that does not add reproducible information beyond conventional risk factors should not be promoted as a clinically useful SMMI biomarker or basis for risk-adapted surveillance.

**Box 2** summarizes the minimum reporting standards for contamination-aware, comparator-defined, and spatially registered studies conducted within the SMMI framework.

## 10 From spatial association to causal and clinical claims

A candidate SMMI microdomain is a spatial nomination, not a demonstrated causal mechanism. **Figure 2** and **Box 1** summarize the evidence ladder: spatial association can prioritize where microbe–host interactions may occur, but temporal evidence and targeted perturbation are needed to strengthen causal interpretation. Model-only results remain biological plausibility unless they are concordant with human OPMD spatial and outcome evidence (Uitto et al., 2005; Bhatia and Ingber, 2014; Gallimidi et al., 2015; Sztukowska et al., 2016; Geng et al., 2017; Lee et al., 2017; Drost and Clevers, 2018; Rahimi et al., 2018; Driehuis et al., 2019; Sellaeg et al., 2024).

**Figure 2:**
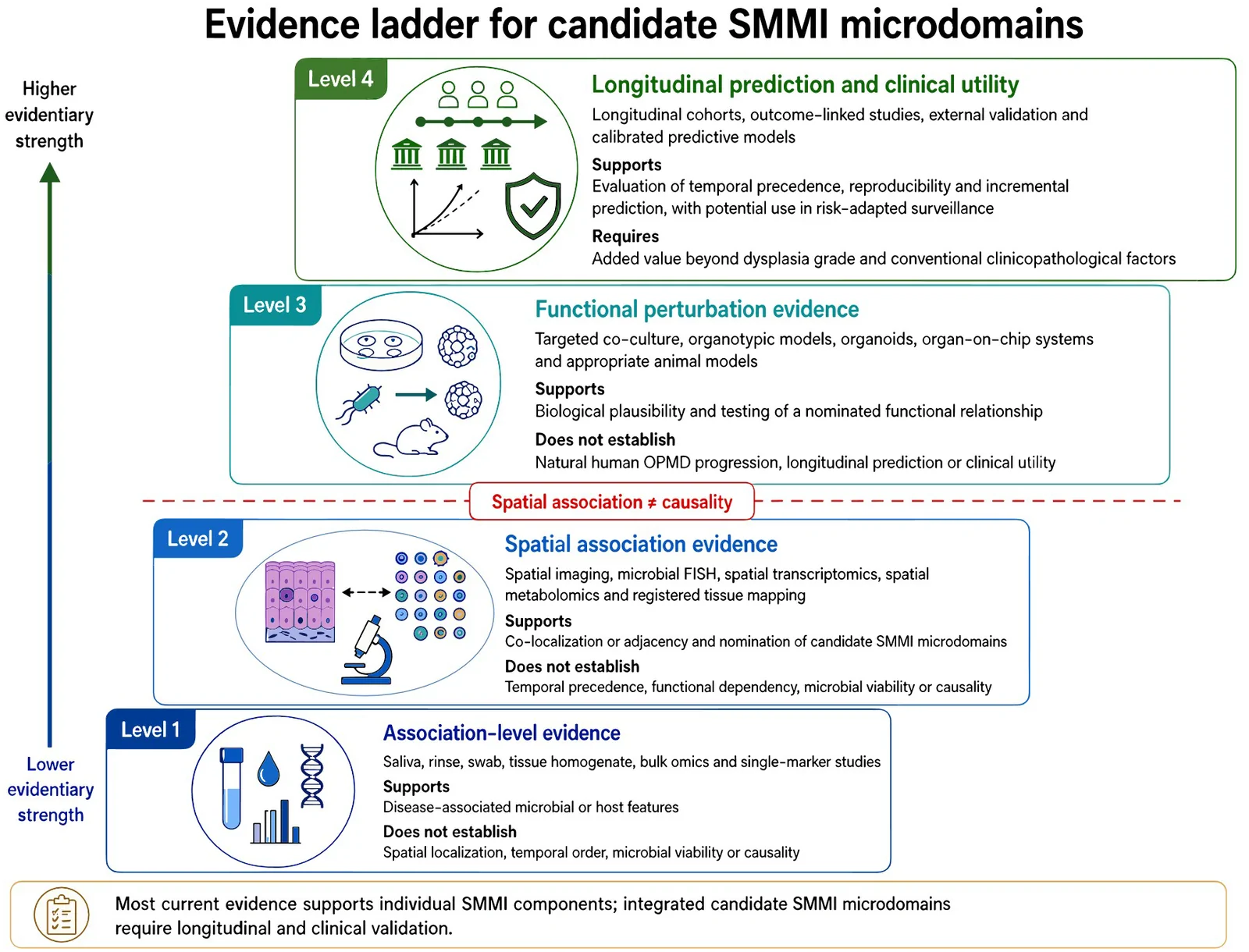
Four-level evidence ladder for candidate SMMI microdomains. The framework separates increasingly demanding forms of evidence needed to move from microbial or host-feature nomination toward clinically useful SMMI readouts. Level 1, association-level evidence, includes bulk tissue, saliva, rinse, swab, or omics studies that identify candidate microbial, epithelial, immune, metabolic, or stromal correlates but cannot localize interactions. Level 2, spatial association evidence, requires microbial signals to be registered with host tissue states by methods such as microbial FISH, RNAscope-like assays, spatial transcriptomics, spatial metabolomics, serial-section mapping, or multiplex imaging. Level 3, functional perturbation evidence, tests whether nominated microbe-host, metabolite-host, or immune-stromal relationships alter relevant phenotypes in controlled systems. Level 4, longitudinal prediction and clinical utility, evaluates temporal order, reproducibility, outcome prediction, and added value beyond conventional pathology. Spatial association can prioritize mechanisms for testing, but does not by itself establish causality, viability, activity, or clinical readiness. This staged structure prevents premature interpretation of candidate microdomains as clinically actionable findings.

Clinical claims require a separate standard. A spatial microbial, metabolic, immune, or epithelial feature should not be treated as a clinically useful biomarker until it shows reproducibility, interpretability, external validation, and incremental value beyond dysplasia grade and conventional factors (Mahmood et al., 2023; Shephard et al., 2024; Ge et al., 2025). Section 9 provides the modular validation route; this section serves only as the bridge from candidate spatial association to causal and clinical claims. The overall modular validation workflow is summarized in **Figure 3**.

**Figure 3:**
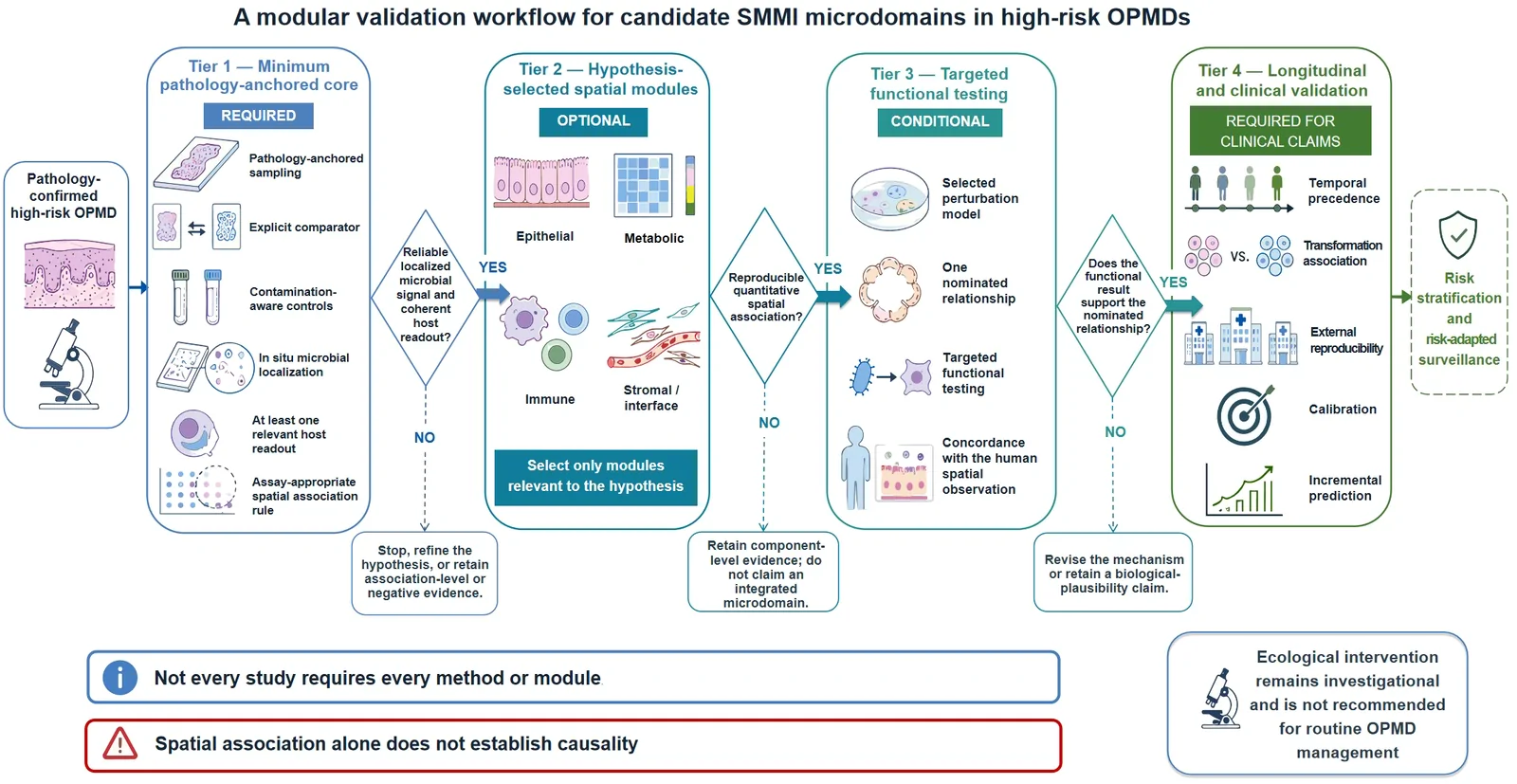
Four-tier modular workflow for SMMI-informed risk stratification and validation in high-risk OPMDs. The workflow begins with Tier 1, a minimum pathology-anchored core that defines lesion phenotype, histopathology, anatomical site, comparator strategy, periodontal context, microbial controls, and spatial registration. Decision nodes then determine whether a candidate spatial association is credible enough to justify deeper testing, whether optional modules are needed, and whether the evidence is sufficient for longitudinal or clinical evaluation. Tier 2 uses hypothesis-selected spatial modules rather than a mandatory all-platform design. Epithelial, immune, metabolic, and stromal/interface modules can be run in parallel according to specimen availability and the biological question. Tier 3 prioritizes targeted functional testing of the most informative candidate microdomains, including microbe-epithelium, metabolite-immune, or stromal-interface relationships. Tier 4 evaluates longitudinal prediction, reproducibility, and clinical utility. YES/NO branches preserve decision discipline by allowing studies to return for refinement when localization, module selection, or functional support is inadequate. The intended output is risk stratification and risk-adapted surveillance, not immediate ecological intervention. This modular design keeps the framework feasible for scarce OPMD specimens while preserving escalation to deeper spatial association, perturbation, and clinical validation only when the preceding tier supports it.

## 11 Translational boundary and concluding perspective: risk stratification before ecological intervention

### 11.1 Near-term translation: risk stratification and surveillance

The near-term translational emphasis of SMMI research is risk stratification and risk-adapted surveillance, not immediate ecological intervention. A practical risk model would likely use a small set of reproducible spatial readouts rather than broad multi-omics panels, and those readouts must add predictive value beyond dysplasia grade and standard clinicopathological factors. Such a model could eventually help identify lesions needing closer surveillance, repeat biopsy, spatial molecular assessment, or intensified follow-up, but only after reproducibility and outcome linkage are demonstrated.

### 11.2 Translational and intervention boundaries

Mechanistic validation and clinical utility remain related but distinct goals. Candidate readouts must be technically robust in clinically available tissue, including FFPE specimens where possible; microbial signals require contamination-aware validation; spatial features must be reproducible across observers, platforms, institutions, and lesion types; and predictive models must undergo external validation. Current evidence does not justify antimicrobial, probiotic, antifungal, immune-modulatory, or metabolic interventions to alter SMMI features for cancer prevention outside research contexts (Hong et al., 1986; Tsao et al., 2009; Lodi et al., 2016; William et al., 2016). Because oral microbial communities also support barrier function, periodontal ecology, immune tolerance, and colonization resistance, intervention risk should be evaluated before any preventive strategy is proposed.

### 11.3 Concluding perspective

This Review proposes the SMMI framework as an evidence-ranked conceptual and validation framework for studying malignant transformation risk in high-risk OPMDs. The current evidence supports individual components, including microbial dysbiosis, impaired epithelial differentiation, transformation-linked metabolic remodeling, macrophage-centered immune remodeling, and candidate epithelial immune-visibility changes, but not a fully validated integrated causal microdomain. The next phase should prioritize lesion-anchored spatial validation, targeted functional testing, and longitudinal clinical validation. By framing high-risk OPMDs as spatially organized oral microbe–host ecosystems, the framework moves beyond taxon-centered dysbiosis and bulk association toward pathology-anchored spatial ecology while preserving the boundary between candidate biology and clinical action.
