# Supplementary material for: A spatial–microbial–metabolic–immune framework for high-risk oral potentially malignant disorders: an evidence-ranked review for malignant transformation risk stratification
Source: Front Cell Infect Microbiol. 2026 Sep 15;16:1928595. doi: 10.3389/fcimb.2026.1928595 (PMC13625268; doi:10.3389/fcimb.2026.1928595)
Supplement: Supplementary file 1 [file Table1.docx]

**Supplementary Tables**

Supplementary tables supporting the spatial–microbial–metabolic–immune framework in high-risk oral potentially malignant disorders

These supplementary tables support the evidence-ranked spatial–microbial–metabolic–immune (SMMI) framework described in the main Review. They distinguish candidate SMMI microdomains and component-level readouts from established causal mechanisms or validated clinical biomarkers. Experimental and spatial platforms are intended to be selected in a modular and hypothesis-driven manner; no single study is expected to apply every method or module. Spatial association encompasses co-localization and adjacency assessed using assay-appropriate analytical rules. Risk stratification and risk-adapted surveillance remain the near-term translational endpoint, whereas ecological intervention remains investigational.

# **Supplementary Table S1. Metabolic readouts and evidence boundaries for microbe-linked biochemical mediation in SMMI research.**

| **Metabolic readout / axis** | **Potential biological source(s)** | **Microbe–host relevance in SMMI research** | **Evidence class / current support** | **Preferred spatial or orthogonal validation** | **Evidence boundary / interpretive caveat** | **Priority next step** |
| --- | --- | --- | --- | --- | --- | --- |
| Lipid remodeling, including phospholipids, oxidized lipids, cholesteryl esters and fatty acids | Dysplastic epithelium, membrane turnover, inflammatory cells, stromal cells, saliva, diet, periodontal inflammation and microbial communities | May reflect epithelial stress adaptation, barrier remodeling, inflammatory mediator production and membrane-state changes near biofilm–barrier interfaces. | Direct human OPMD association for selected metabolomic signatures; broader cancer biology for functional interpretation. | Spatial metabolomics registered to histopathology, microbial localization, epithelial differentiation markers and immune-cell imaging. | Bulk lipid changes do not establish microbial source, local gradient, temporal order or functional dependency. | Compare transforming versus non-transforming OPMDs and test whether lipid readouts add value beyond dysplasia grade and clinicopathological factors. |
| Oxidative-stress products and redox imbalance | Epithelial stress, inflammatory cells, microbial stimulation, tobacco/alcohol/areca exposure, periodontal inflammation and stromal remodeling | May mediate links between microbial irritation, epithelial DNA damage, repair programs and altered immune visibility. | Preclinical and broader oral carcinogenesis evidence; limited direct spatial human OPMD evidence. | Co-map oxidative-stress markers with microbial probes, DNA-damage markers, epithelial differentiation and macrophage/T-cell positioning. | Oxidative stress is non-specific and may arise from exposures or inflammation independent of microbial contribution. | Use lesion-directed sampling with exposure and periodontal covariates; prioritize registered spatial analysis rather than isolated marker abundance. |
| Acetaldehyde-related biochemical stress | Alcohol metabolism, Candida and bacterial metabolism, epithelial metabolism and local microbial ecology | May connect microbial/fungal activity, alcohol exposure, epithelial genotoxic stress and mucosal irritation in selected lesions. | Biological plausibility and observational support; evidence remains context-dependent. | Combine Candida/bacterial localization, exposure history, acetaldehyde-related readouts, epithelial DNA damage and lesion outcome data. | Association with acetaldehyde-related stress does not prove that microbes initiate transformation. | Interpret as a candidate mediation axis requiring source attribution, spatial localization and longitudinal validation. |
| Lactate-related and glycolysis/hypoxia-associated readouts | Dysplastic epithelium, stromal cells, inflammatory cells, microbial metabolism and hypoxic tissue regions | May link epithelial stress adaptation, stromal remodeling and immune-cell dysfunction in candidate microdomains. | Broader cancer immunometabolism provides plausibility; direct OPMD spatial evidence is weak. | Spatial metabolomics or hypoxia/glycolysis marker imaging registered with CD8+ T-cell distance, macrophage states and microbial localization. | Broader cancer evidence should not be used as direct evidence that lactate gradients drive immune restriction in human OPMDs. | Prioritize lactate/hypoxia readouts only when co-registered with host–microbe and immune spatial features. |
| Tryptophan–kynurenine pathway and IDO1-associated signals | IDO1-positive macrophages, epithelial cells, dendritic cells, stromal cells and inflammatory microenvironments | Connects immunometabolism with macrophage-centered immunoregulation and potential T-cell restriction in high-risk lesions. | Direct human OPMD evidence for IDO1-positive macrophage association; broader pathway evidence for kynurenine-mediated immunoregulation. | Co-map IDO1+ macrophages, kynurenine-related metabolites, CD8+ T-cell distance, Treg localization and microbial signals. | IDO1 enrichment does not prove local kynurenine accumulation, microbial induction or T-cell suppression. | Use spatial metabolomics, multiplex immune imaging and perturbation systems to test macrophage–metabolite–T-cell relationships. |
| Short-chain-fatty-acid- or fermentation-related signals | Bacterial fermentation, diet, saliva, plaque, periodontal pockets and epithelial uptake/metabolism | May influence barrier integrity, epithelial differentiation, inflammatory tone and immune-cell function depending on local concentration and context. | Strong biological plausibility in mucosal biology; limited direct human OPMD spatial evidence. | Measure local metabolite distribution with microbial community mapping and epithelial/immune response markers. | Saliva or bulk tissue levels cannot define local source, concentration or functional direction. | Treat as a hypothesis-generating readout unless spatially resolved and outcome-linked. |
| Proteolytic activity and matrix-degradation-related biochemical signals | Periodontal/pathobiont-associated microbes, epithelial cells, macrophages, fibroblasts and extracellular matrix remodeling | May connect biofilm ecology, epithelial injury, stromal remodeling and wound-repair-like host responses. | Preclinical and broader oral inflammatory disease evidence; direct OPMD transformation evidence limited. | Register protease activity or matrix-remodeling markers with microbial localization, epithelial barrier disruption and stromal immune neighborhoods. | Proteolytic activity may reflect inflammation or periodontal disease rather than lesion-specific transformation biology. | Control for periodontal status, plaque burden and site; require lesion-localized validation. |
| Eicosanoid or inflammatory lipid mediator signals | Epithelial cells, macrophages, neutrophils, fibroblasts and inflammatory microenvironments | May link microbial stimulation with inflammatory tone, epithelial repair and immune-cell recruitment. | Broader inflammation and cancer biology; limited direct SMMI-specific OPMD evidence. | Spatial lipidomics or immunostaining for pathway components registered with macrophage/T-cell architecture and microbial localization. | Inflammatory lipid mediators are not microbe-specific and cannot define an SMMI microdomain alone. | Use as supporting host-response readouts within multi-layer spatial models, not standalone biomarkers. |
| Polyamine, amino-acid and nucleotide-turnover-related signals | Proliferating epithelium, microbes, immune cells and tissue repair programs | May reflect epithelial proliferation, microbial metabolism, oxidative stress and tissue repair in high-risk fields. | Mostly broader metabolism biology and association-level evidence. | Co-register with proliferation, DNA damage, microbial localization and immune-cell spatial readouts. | Turnover-related metabolites are highly non-specific and should not be interpreted as microbial mechanisms without source attribution. | Include only when analytically robust and interpreted within a registered host–microbe spatial context. |

Note: IDO1, indoleamine 2,3-dioxygenase 1; OPMD, oral potentially malignant disorder; SMMI, spatial–microbial–metabolic–immune; Treg/Tregs, regulatory T cell(s). These readouts are candidate mediation layers. They should not be interpreted as causal axes without spatial registration, temporal evidence, perturbation testing and outcome linkage.

# **Supplementary Table S2. Hypothesis-selected experimental and validation platforms for candidate SMMI relationships.**

| **Experimental platform** | **Best-suited question** | **Candidate SMMI interaction tested** | **Core readouts** | **Strengths** | **Limitations / caveats** | **Minimum controls and reporting** | **Evidence contribution** | **Role in the modular roadmap** |
| --- | --- | --- | --- | --- | --- | --- | --- | --- |
| Oral keratinocyte monolayer co-culture with candidate microbes | Does a nominated microbe or microbial product alter epithelial barrier-response states? | Microbe–epithelium interaction, inflammatory signaling, differentiation change and stress response. | KRT4/KRT13/cornulin, KRT5/KRT14/TP63, TLR/NF-κB response, IL-1β/IL-6/IL-8, DNA damage and viability. | Controlled exposure, low cost, mechanistic tractability and dose/time testing. | Limited stratification, simplified microbiota, altered oxygen tension and weak stromal/immune context. | Report species/strain, MOI or inoculum, viability, anaerobic/aerobic conditions, exposure duration, antibiotics, cytotoxicity and negative/heat-killed controls. | Functional perturbation evidence; does not model natural OPMD progression. | Tier 3 conditional functional testing |
| Primary oral epithelial cells or dysplastic epithelial cell models | Are responses preserved in less transformed or lesion-relevant epithelial backgrounds? | Barrier injury, differentiation impairment, epithelial immune visibility and microbial-response programs. | Differentiation markers, HLA class I/β2-microglobulin/TAP/NLRC5, interferon-response genes, cytokines, DNA damage and wound-repair-like programs. | More relevant than established OSCC cell lines when available. | Limited availability, donor variability, finite lifespan and batch effects. | Report donor/lesion source, passage, differentiation state, mycoplasma status and exposure conditions. | Strengthens biological plausibility but still requires human tissue validation. | Tier 3 conditional functional testing |
| Air-liquid interface or organotypic oral mucosa models | Does stratification modify microbe–epithelium responses? | Biofilm–barrier interface, epithelial differentiation, permeability, antimicrobial response and wound-repair-like adaptation. | Barrier integrity, stratification markers, microbial localization, cytokines, antimicrobial peptides and tissue architecture. | Better preserves epithelial layering and surface exposure than monolayers. | Usually lacks full immune, vascular and stromal complexity; microbial exposure may be artificial. | Report tissue construction, differentiation time, surface colonization method, microbial burden and histological quality. | Intermediate functional model for spatially informed hypotheses. | Tier 3 conditional functional testing |
| Oral mucosal organoids or epithelial organoids | Can lesion- or patient-derived epithelial states show differential microbial or metabolite responses? | Epithelial susceptibility, differentiation state, stress tolerance and immune-visibility programs. | Growth, differentiation, transcriptomic response, antigen-presentation markers, DNA damage and metabolite response. | Patient-specific modeling and repeated perturbation are possible. | May lack native surface polarity, biofilm interface, stromal structure and immune context unless engineered. | Report derivation, passage, culture matrix, differentiation protocol, organoid polarity and validation against source tissue. | Functional plausibility and patient-specific hypothesis testing. | Tier 3 conditional functional testing |
| Organoid–immune–microbe co-culture | Can microbial or metabolic perturbation change macrophage/T-cell interactions with epithelial states? | Microbe–epithelium–macrophage or metabolite–immune interactions, including IDO1 and T-cell restriction hypotheses. | IDO1+ macrophages, cytokines, antigen-presentation markers, CD8+ T-cell activation/distance proxies, Treg markers and epithelial stress. | Allows multi-cell testing of prioritized SMMI interactions. | Technically complex, sensitive to culture conditions and not fully spatially equivalent to tissue. | Report immune-cell source, activation state, ratios, co-culture duration, microbial viability and controls without each component. | Functional perturbation evidence for mechanistic dependency. | Tier 3 conditional functional testing |
| Microfluidic oral mucosa-on-chip or organ-on-chip platform | Can dynamic exposure, gradients or barrier flow modulate host–microbe responses? | Biofilm–barrier gradients, metabolite gradients, immune migration and epithelial-stromal crosstalk. | Barrier permeability, live imaging, microbial localization, cytokines, immune-cell migration and spatial gradients. | Enables controlled microenvironment, flow, gradients and repeated sampling. | Requires specialized setup; throughput and biological complexity vary. | Report chip design, flow rate, oxygen conditions, surface coating, microbial inoculum and reproducibility metrics. | Advanced functional model; supports but does not replace human OPMD validation. | Tier 3 conditional functional testing |
| Macrophage or T-cell immune co-culture systems | Do candidate microbes or metabolites alter immune states relevant to host-response restriction? | Macrophage-centered immunoregulation, T-cell activation/suppression, IDO1–kynurenine and checkpoint-associated readouts. | IDO1, CD68/CD163/CD206, cytokines, kynurenine pathway markers, CD8+ T-cell activation, exhaustion markers and cytotoxicity. | Tests immune mechanisms directly and can be paired with epithelial readouts. | Immune cells may differ from tissue-resident oral mucosal populations; spatial architecture is simplified. | Report immune-cell source, polarization/activation conditions, microbial/metabolite doses and viability/cytotoxicity controls. | Functional evidence for immune component of candidate SMMI interactions. | Tier 3 conditional functional testing |
| Defined metabolite perturbation assays | Can candidate metabolites affect epithelial stress, differentiation or immune response? | Lactate/hypoxia, kynurenine/IDO1, acetaldehyde-related stress, oxidative stress and lipid mediator hypotheses. | Epithelial differentiation, DNA damage, antigen presentation, cytokines, macrophage state, T-cell activation and cell viability. | Mechanistically focused and dose-responsive. | Physiologic concentration, local source and tissue gradient may be uncertain. | Use biologically plausible concentrations, pH/osmolarity controls, time courses and toxicity controls. | Perturbation support for candidate mediation axes. | Tier 3 conditional functional testing |
| Ex vivo lesion tissue, FFPE serial-section validation or fresh tissue explants | Are nominated interactions visible in lesion tissue context? | Spatial association assessment of microbial, epithelial, metabolic, stromal and immune layers. | Microbial FISH/RNAscope-like signals, epithelial markers, spatial metabolomics, multiplex immune imaging and histopathology. | Maintains native architecture and links to clinicopathological annotation. | Limited perturbation potential; FFPE may limit viability/activity inference. | Report section order, controls, pathology registration, segmentation method, spatial resolution and uncertainty. Report pathology-defined compartment, explicit comparator, contamination-aware controls, distance or neighborhood rule, assay resolution, registration uncertainty, selected spatial metric, comparator or null model, and threshold rationale. | Spatial nomination and validation; functional inference remains limited. | Tier 1 core or Tier 2 optional module; microbial FISH/RNAscope-like localization is Tier 1 core when microbial localization is claimed; spatial metabolomics is a Tier 2 optional metabolic module |
| 4NQO oral carcinogenesis model | Do candidate host-response, immune or microbial perturbations affect lesion development over time? | Temporal relationships among epithelial injury, inflammation, microbial ecology, macrophage states and lesion progression. | Lesion burden, dysplasia/OSCC transition, immune states, microbial shifts, epithelial markers and spatial imaging. | Provides temporal and perturbation context. | Murine anatomy, microbiota, carcinogen exposure and immune kinetics differ from human OPMDs. | Report strain, sex, dose, exposure duration, oral site, microbiota conditions and blinded histopathology. | Preclinical perturbation evidence; not direct proof of human OPMD progression. | Tier 3 conditional preclinical testing |
| Microbiome-modified, antibiotic-treated or gnotobiotic animal models | Does changing microbial ecology alter lesion-associated host responses? | Community-level microbial contribution to epithelial stress, inflammation, metabolism and immunity. | Microbial composition, lesion outcome, barrier markers, cytokines, immune-cell positioning and metabolite readouts. | Tests ecological contribution and reversibility. | Antibiotics or colonization may have broad off-target effects; translation to human oral ecology is limited. | Report microbiome manipulation, colonization confirmation, off-target effects, housing, diet and longitudinal sampling. | Preclinical ecological plausibility; clinical intervention claims require much stronger evidence. | Tier 3 conditional ecological plausibility testing |

Platforms should be selected according to the biological hypothesis, specimen availability, assay resolution, and preceding human spatial evidence. The table does not imply that all platforms are required within a single study. Tier 4 longitudinal and clinical validation requires human outcome-linked cohorts and is not replaced by functional or animal models. Comparator hierarchy should be prespecified as: within-lesion reference; patient-matched reference mucosa; and external matched healthy control. Adjacent normal-appearing mucosa should be treated as patient-matched reference tissue rather than an unequivocally healthy baseline because field effects may persist. Abbreviations: 4NQO, 4-nitroquinoline 1-oxide; B2M, β2-microglobulin; FFPE, formalin-fixed, paraffin-embedded; FISH, fluorescence in situ hybridization; IDO1, indoleamine 2,3-dioxygenase 1; MOI, multiplicity of infection; NF-κB, nuclear factor-κB; OPMD, oral potentially malignant disorder; SMMI, spatial–microbial–metabolic–immune; TLR, Toll-like receptor; Tregs, regulatory T cells.

# **Supplementary Table S3. Translational readiness and validation requirements for SMMI components.**

| **SMMI component / candidate readout** | **Current translational role** | **Approximate readiness** | **Minimum validation requirements** | **Clinical utility benchmark** | **Major confounders / risks** | **Recommended near-term use** |
| --- | --- | --- | --- | --- | --- | --- |
| Taxon-level oral microbial dysbiosis signatures | Association-level risk context and hypothesis generation. | Low to moderate; signatures vary by cohort, sampling source and confounding. | Lesion-directed sampling, periodontal and exposure covariates, contamination controls, reproducibility across cohorts and spatial validation. | Must improve prediction beyond dysplasia grade, lesion phenotype, site, smoking/alcohol/areca exposure and periodontal status. | Periodontal disease, plaque, tooth proximity, saliva contamination, antibiotics/antifungals, oral hygiene and batch effects. | Use as candidate microbial context, not as a standalone clinical biomarker. |
| Contamination-aware microbial localization | Spatial nomination of microbial signals in lesion tissue context. | Moderate for selected organisms when orthogonal controls are used. | Microbial FISH/RNAscope-like validation, negative/positive probe controls, section-order controls, reagent controls and host-marker co-registration. | Should identify reproducible lesion-localized signals associated with high-risk histology or transformation outcome. | Low biomass, paraffin processing, reagent contaminants, probe cross-reactivity and dead/extracellular DNA. | Prioritize for spatial validation studies and tissue-based hypothesis testing. |
| Epithelial differentiation and barrier-response markers | Host substrate layer for risk interpretation and spatial registration. | Moderate to high for epithelial-risk context; lower for microbe–host coupling. | Co-register KRT4/KRT13/cornulin and basal/progenitor markers with microbial localization, metabolic readouts and immune-cell positioning. | Should add spatial context beyond conventional dysplasia grading and improve lesion-level risk models. | Sampling heterogeneity, inflammatory repair, keratinization, fixation effects and interobserver pathology variation. | Use as host epithelial anchor, not as proof of an SMMI microdomain alone. |
| Clonal field-risk or genomic instability readouts | Host susceptibility layer and risk covariate. | Moderate for risk biology; low for direct SMMI coupling. | Integrate with histopathology, spatial host-response markers, microbial localization and longitudinal outcome data. | Should stratify lesions or fields with incremental value beyond morphology and clinical phenotype. | Field heterogeneity, biopsy sampling, lesion recurrence, exposure history and assay platform differences. | Use to contextualize host susceptibility; avoid presenting as microbe-associated evidence unless spatially linked. |
| Metabolic readouts, including lipid remodeling, lactate/hypoxia and kynurenine-related axes | Candidate biochemical mediation layer linking microbial ecology, epithelium and immunity. | Low to moderate; strongest as association-level or hypothesis-generating evidence. | Spatial metabolomics, source attribution, registered microbial/immune imaging, perturbation testing and longitudinal validation. | Should show reproducible localization and predictive contribution beyond epithelial and immune features. | Mixed tissue sources, diet, saliva, periodontal inflammation, necrosis, platform variability and annotation uncertainty. | Use as candidate mediation readouts; do not treat as causal metabolic axes without functional validation. |
| IDO1-positive macrophages and macrophage polarization | Candidate immunometabolic host-response layer associated with leukoplakia transformation. | Moderate; one of the stronger direct human OPMD-related immune components. | Multiplex immune imaging, spatial proximity to dysplastic epithelium and microbial/metabolic readouts, validation in independent longitudinal cohorts. | Should improve prediction of transformation or high-risk status beyond dysplasia grade and clinical covariates. | Inflammation, lesion site, ulceration, periodontal disease, staining thresholds and macrophage-marker specificity. | Prioritize as a core immune readout for SMMI validation studies. |
| CD8+ T-cell distance, Treg localization and immune spatial neighborhoods | Spatial host-response restriction and immune-positioning readout. | Low to moderate; promising but requires standardized spatial analytics. | Multiplex immune imaging, epithelial segmentation, distance metrics, macrophage/Treg co-localization and outcome-linked cohorts. | Should define reproducible immune-neighborhood features associated with transformation risk or surveillance intensity. | Inflammation, tissue orientation, section thickness, segmentation error and threshold selection. | Use as spatial immune architecture readout, not as isolated abundance. |
| Epithelial immune-visibility markers, including HLA class I, β2-microglobulin, TAP1/2, NLRC5 and interferon-response markers | Candidate epithelial immune-engagement layer. | Low to moderate; direct OPMD evidence remains limited and OSCC-derived inference should be labeled. | Co-map epithelial-state markers, antigen-presentation markers, CD8+ T-cell distance, checkpoint markers, microbial localization and outcome data. | Should identify lesions with altered immune engagement and incremental risk information. | OSCC-to-OPMD extrapolation, inflammatory induction, antibody specificity and epithelial/stromal segmentation. | Use conservatively as candidate immune-visibility readouts requiring OPMD-specific validation. |
| Integrated candidate SMMI microdomain or composite SMMI risk score | Research-stage integrative risk model. | Low; integrated candidate SMMI microdomains remain a Level 4 validation goal. | Multicenter lesion cohorts, standardized sampling, registered spatial omics/imaging, independent validation, calibration and decision-curve analysis. Integrated candidate SMMI microdomains remain a Level 4 validation goal. | Must demonstrate external validation, calibration, incremental prediction, and added value beyond dysplasia grade and conventional clinicopathological factors for risk stratification and risk-adapted surveillance. | Overfitting, platform heterogeneity, missing covariates, lesion heterogeneity and insufficient transformation events. | Develop as an interpretable research model before clinical deployment. |
| Ecological intervention targeting microbial, fungal, immune or metabolic SMMI features | Future research-only possibility. | Not ready for routine OPMD management. | Requires causal evidence, target specificity, safety, reversibility, durability, microbiome-resistance assessment and clinical benefit. | Would need to reduce transformation risk or improve clinically meaningful outcomes without harming oral barrier ecology. | Microbial dysbiosis is context-dependent; interventions may affect barrier function, periodontal health, immune tolerance and resistance. | Not ready for routine OPMD management; do not recommend antimicrobial, probiotic, antifungal, immune or metabolic intervention as current routine OPMD cancer-prevention strategy outside research contexts. |

Note: Readiness categories are qualitative and intended to guide research prioritization. The near-term clinical endpoint for SMMI research is risk stratification and risk-adapted surveillance, not ecological intervention. Abbreviations: B2M, β2-microglobulin; FFPE, formalin-fixed, paraffin-embedded; FISH, fluorescence in situ hybridization; IDO1, indoleamine 2,3-dioxygenase 1; OPMD, oral potentially malignant disorder; SMMI, spatial–microbial–metabolic–immune; Treg/Tregs, regulatory T cell(s).
